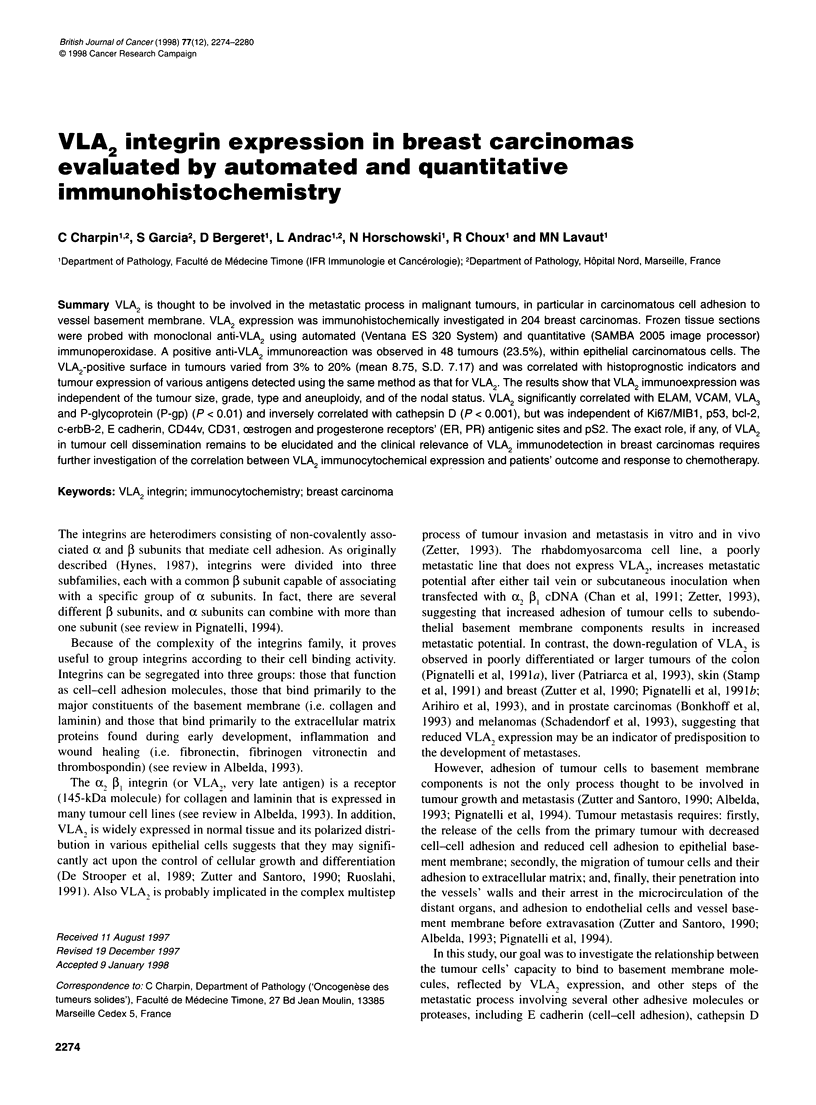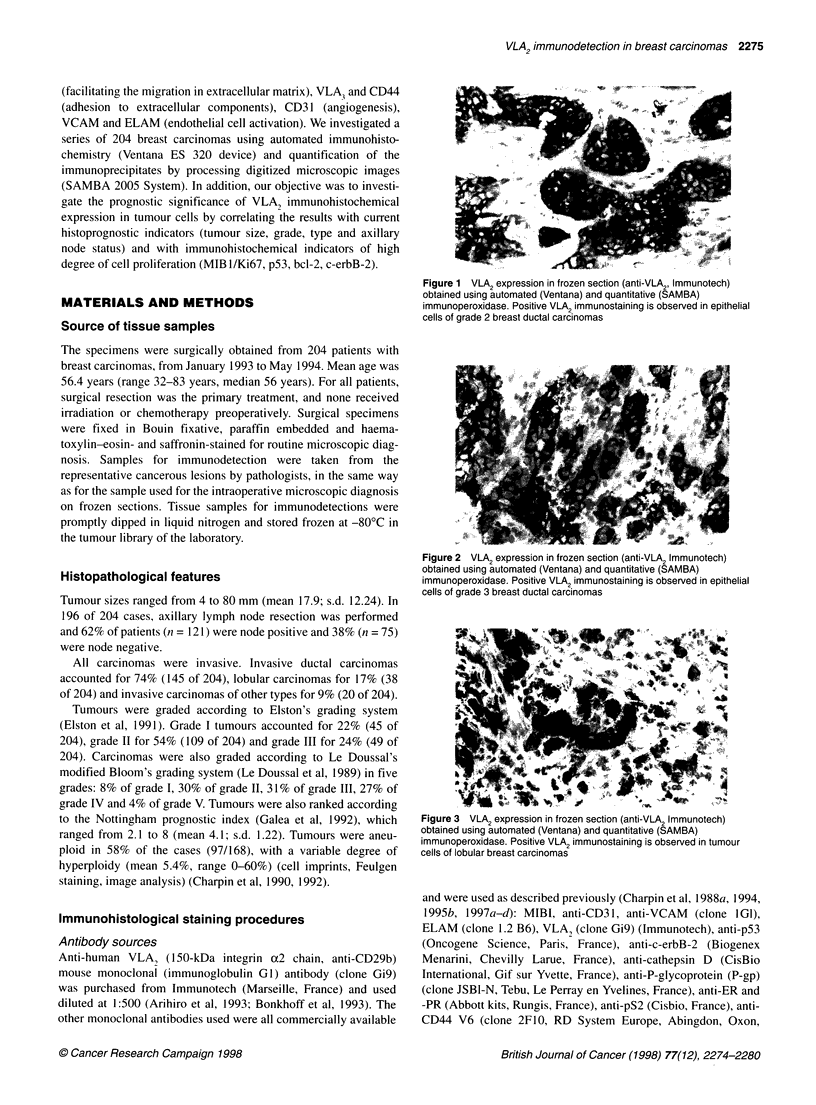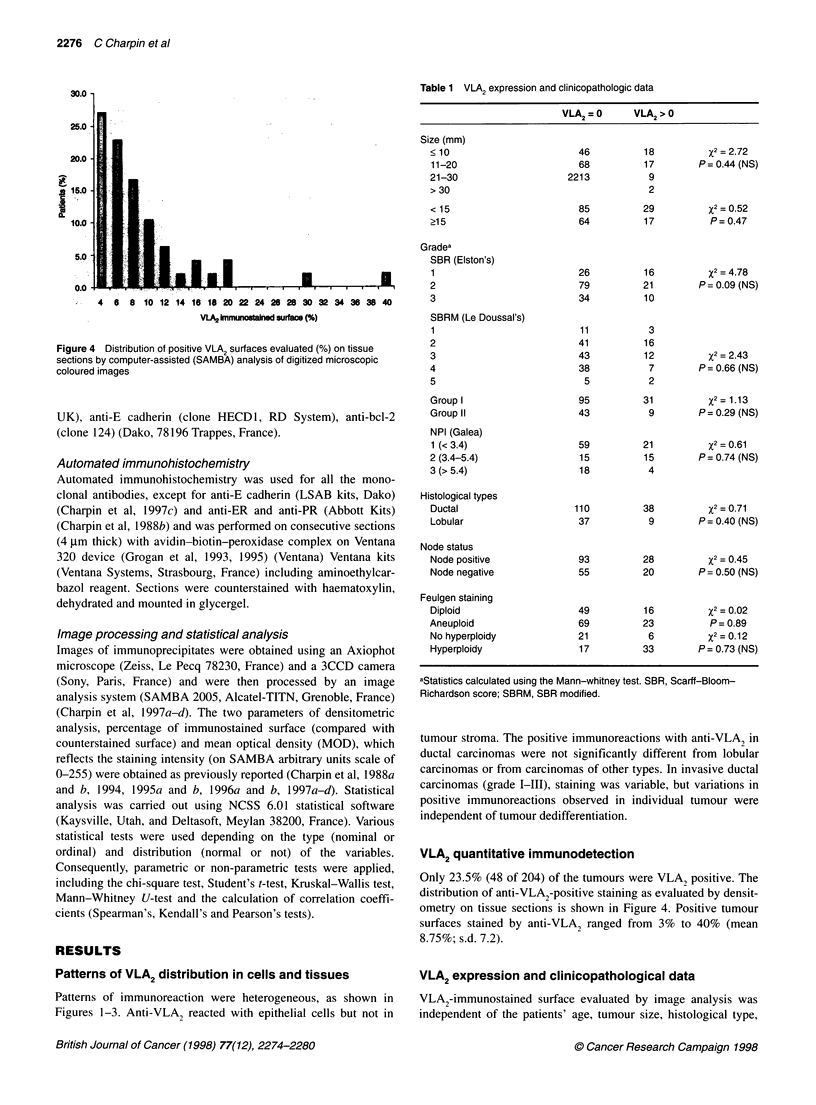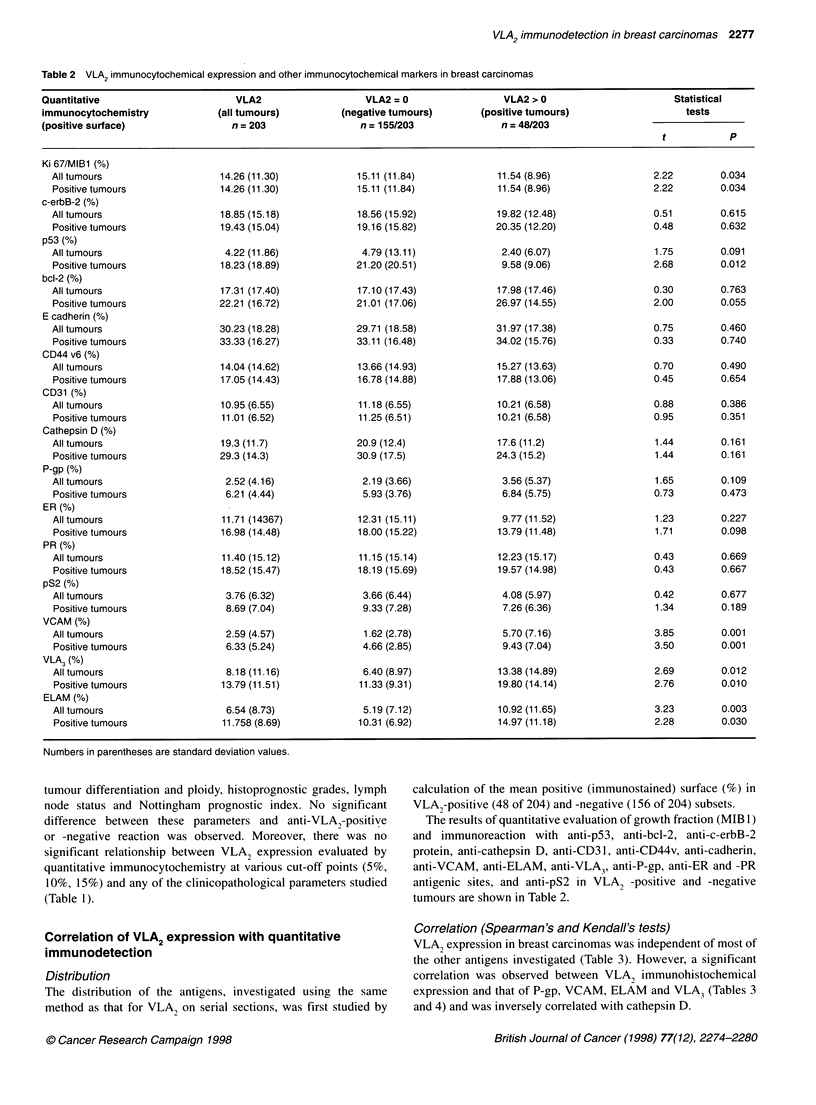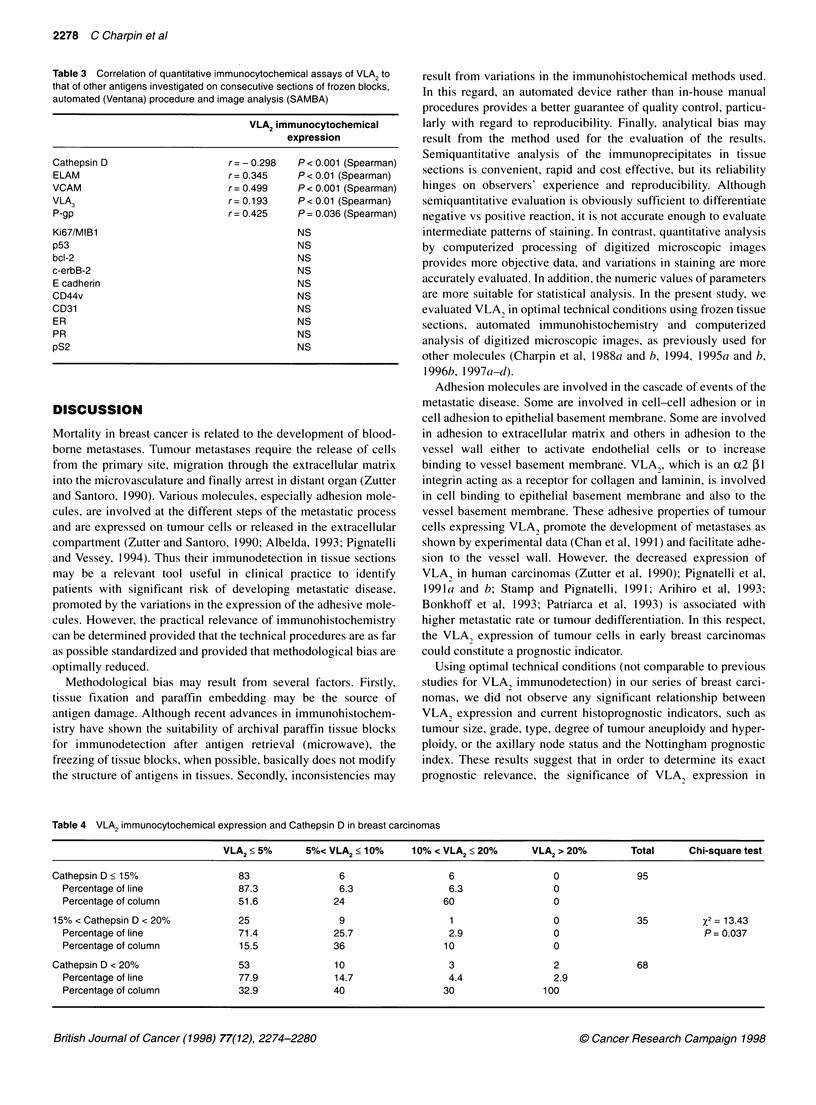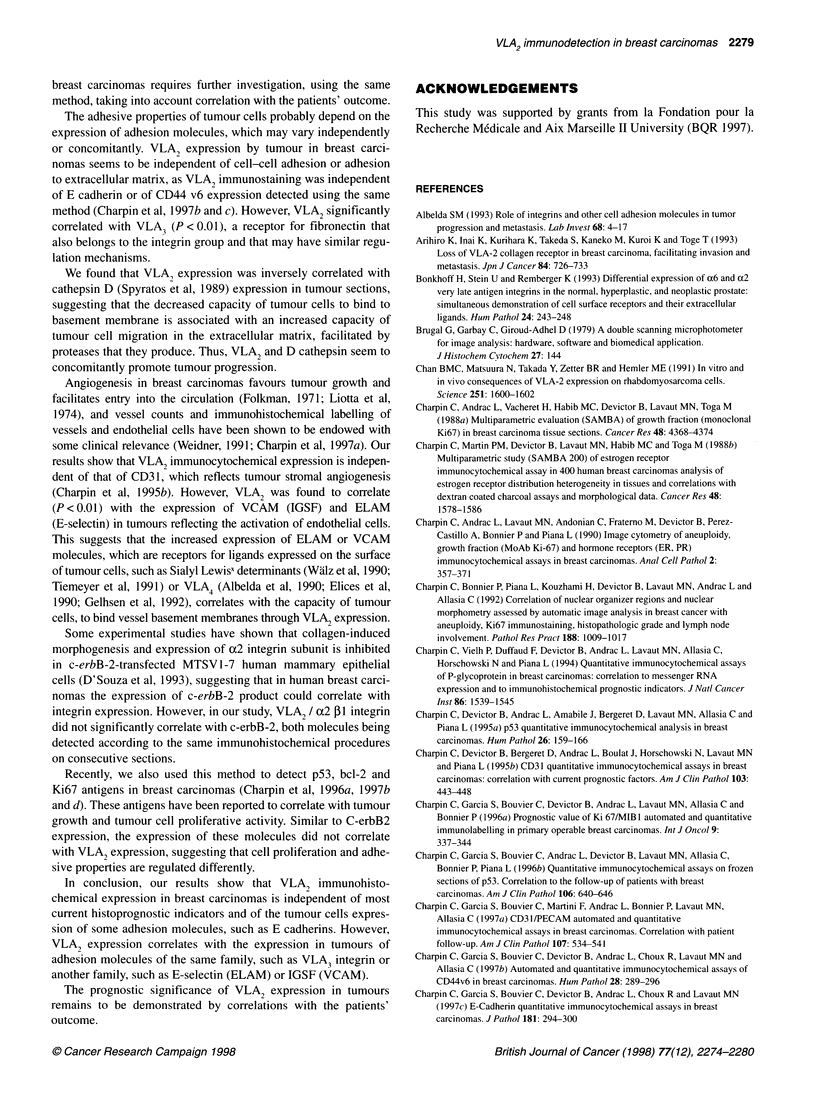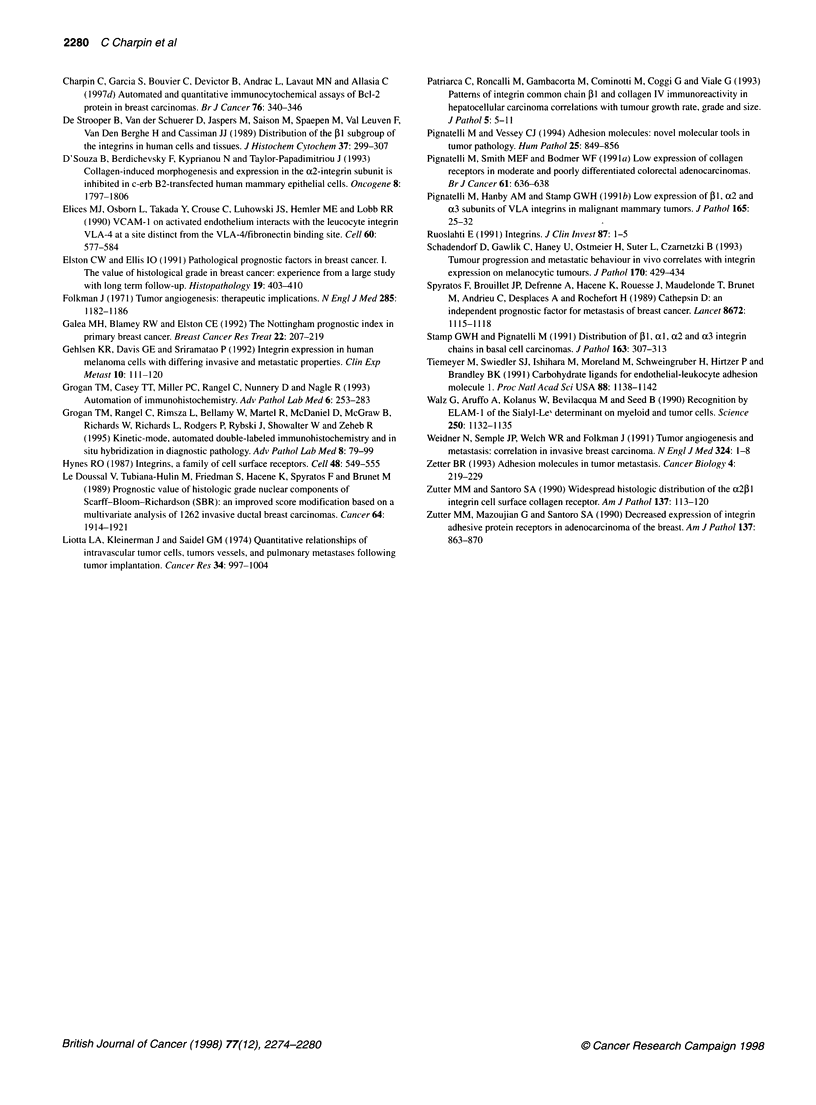# VLA2 integrin expression in breast carcinomas evaluated by automated and quantitative immunohistochemistry.

**DOI:** 10.1038/bjc.1998.378

**Published:** 1998-06

**Authors:** C. Charpin, S. Garcia, D. Bergeret, L. Andrac, N. Horschowski, R. Choux, M. N. Lavaut

**Affiliations:** Department of Pathology, FacultÃ© de MÃ©decine Timore (IFR Immunologie et CancÃ©rologie), HÃ´pital Nord, Marseille, France.

## Abstract

**Images:**


British Journal of Cancer (1998) 77(12), 2274–2280
© 1998 Cancer Research Campaign

### VLA$_2$ integrin expression in breast carcinomas evaluated by automated and quantitative immunohistochemistry

**C Charpin[1,2], S Garcia[2], D Bergeret[1], L Andrac[1,2], N Horschowski[1], R Choux[1] and MN Lavaut[1]**

[1]Department of Pathology, Faculté de Médecine Timone (IFR Immunologie et Cancérologie); [2]Department of Pathology, Hôpital Nord, Marseille, France

**Summary** VLA$_2$ is thought to be involved in the metastatic process in malignant tumours, in particular in carcinomatous cell adhesion to vessel basement membrane. VLA$_2$ expression was immunohistochemically investigated in 204 breast carcinomas. Frozen tissue sections were probed with monoclonal anti-VLA$_2$ using automated (Ventana ES 320 System) and quantitative (SAMBA 2005 image processor) immunoperoxidase. A positive anti-VLA$_2$ immunoreaction was observed in 48 tumours (23.5%), within epithelial carcinomatous cells. The VLA$_2$-positive surface in tumours varied from 3% to 20% (mean 8.75, S.D. 7.17) and was correlated with histoprognostic indicators and tumour expression of various antigens detected using the same method as that for VLA$_2$. The results show that VLA$_2$ immunoexpression was independent of the tumour size, grade, type and aneuploidy, and of the nodal status. VLA$_2$ significantly correlated with ELAM, VCAM, VLA$_3$ and P-glycoprotein (P-gp) ($P < 0.01$) and inversely correlated with cathepsin D ($P < 0.001$), but was independent of Ki67/MIB1, p53, bcl-2, c-erbB-2, E cadherin, CD44v, CD31, œstrogen and progesterone receptors' (ER, PR) antigenic sites and pS2. The exact role, if any, of VLA$_2$ in tumour cell dissemination remains to be elucidated and the clinical relevance of VLA$_2$ immunodetection in breast carcinomas requires further investigation of the correlation between VLA$_2$ immunocytochemical expression and patients' outcome and response to chemotherapy.

**Keywords:** VLA$_2$ integrin; immunocytochemistry; breast carcinoma

The integrins are heterodimers consisting of non-covalently associated $\alpha$ and $\beta$ subunits that mediate cell adhesion. As originally described (Hynes, 1987), integrins were divided into three subfamilies, each with a common $\beta$ subunit capable of associating with a specific group of $\alpha$ subunits. In fact, there are several different $\beta$ subunits, and $\alpha$ subunits can combine with more than one subunit (see review in Pignatelli, 1994).

Because of the complexity of the integrins family, it proves useful to group integrins according to their cell binding activity. Integrins can be segregated into three groups: those that function as cell–cell adhesion molecules, those that bind primarily to the major constituents of the basement membrane (i.e. collagen and laminin) and those that bind primarily to the extracellular matrix proteins found during early development, inflammation and wound healing (i.e. fibronectin, fibrinogen vitronectin and thrombospondin) (see review in Albelda, 1993).

The $\alpha_2$ $\beta_1$ integrin (or VLA$_2$, very late antigen) is a receptor (145-kDa molecule) for collagen and laminin that is expressed in many tumour cell lines (see review in Albelda, 1993). In addition, VLA$_2$ is widely expressed in normal tissue and its polarized distribution in various epithelial cells suggests that they may significantly act upon the control of cellular growth and differentiation (De Strooper et al, 1989; Zutter and Santoro, 1990; Ruoslahi, 1991). Also VLA$_2$ is probably implicated in the complex multistep process of tumour invasion and metastasis in vitro and in vivo (Zetter, 1993). The rhabdomyosarcoma cell line, a poorly metastatic line that does not express VLA$_2$, increases metastatic potential after either tail vein or subcutaneous inoculation when transfected with $\alpha_2$ $\beta_1$ cDNA (Chan et al, 1991; Zetter, 1993), suggesting that increased adhesion of tumour cells to subendothelial basement membrane components results in increased metastatic potential. In contrast, the down-regulation of VLA$_2$ is observed in poorly differentiated or larger tumours of the colon (Pignatelli et al, 1991*a*), liver (Patriarca et al, 1993), skin (Stamp et al, 1991) and breast (Zutter et al, 1990; Pignatelli et al, 1991*b*; Arihiro et al, 1993), and in prostate carcinomas (Bonkhoff et al, 1993) and melanomas (Schadendorf et al, 1993), suggesting that reduced VLA$_2$ expression may be an indicator of predisposition to the development of metastases.

However, adhesion of tumour cells to basement membrane components is not the only process thought to be involved in tumour growth and metastasis (Zutter and Santoro, 1990; Albelda, 1993; Pignatelli et al, 1994). Tumour metastasis requires: firstly, the release of the cells from the primary tumour with decreased cell–cell adhesion and reduced cell adhesion to epithelial basement membrane; secondly, the migration of tumour cells and their adhesion to extracellular matrix; and, finally, their penetration into the vessels' walls and their arrest in the microcirculation of the distant organs, and adhesion to endothelial cells and vessel basement membrane before extravasation (Zutter and Santoro, 1990; Albelda, 1993; Pignatelli et al, 1994).

In this study, our goal was to investigate the relationship between the tumour cells' capacity to bind to basement membrane molecules, reflected by VLA$_2$ expression, and other steps of the metastatic process involving several other adhesive molecules or proteases, including E cadherin (cell–cell adhesion), cathepsin D

*Received 11 August 1997*
*Revised 19 December 1997*
*Accepted 9 January 1998*

*Correspondence to:* C Charpin, Department of Pathology ('Oncogenèse des tumeurs solides'), Faculté de Médecine Timone, 27 Bd Jean Moulin, 13385 Marseille Cedex 5, France

(facilitating the migration in extracellular matrix), $VLA_3$ and CD44 (adhesion to extracellular components), CD31 (angiogenesis), VCAM and ELAM (endothelial cell activation). We investigated a series of 204 breast carcinomas using automated immunohistochemistry (Ventana ES 320 device) and quantification of the immunoprecipitates by processing digitized microscopic images (SAMBA 2005 System). In addition, our objective was to investigate the prognostic significance of $VLA_2$ immunohistochemical expression in tumour cells by correlating the results with current histoprognostic indicators (tumour size, grade, type and axillary node status) and with immunohistochemical indicators of high degree of cell proliferation (MIB1/Ki67, p53, bcl-2, c-erbB-2).

### MATERIALS AND METHODS

### Source of tissue samples

The specimens were surgically obtained from 204 patients with breast carcinomas, from January 1993 to May 1994. Mean age was 56.4 years (range 32–83 years, median 56 years). For all patients, surgical resection was the primary treatment, and none received irradiation or chemotherapy preoperatively. Surgical specimens were fixed in Bouin fixative, paraffin embedded and haematoxylin–eosin- and saffronin-stained for routine microscopic diagnosis. Samples for immunodetection were taken from the representative cancerous lesions by pathologists, in the same way as for the sample used for the intraoperative microscopic diagnosis on frozen sections. Tissue samples for immunodetections were promptly dipped in liquid nitrogen and stored frozen at –80°C in the tumour library of the laboratory.

### Histopathological features

Tumour sizes ranged from 4 to 80 mm (mean 17.9; s.d. 12.24). In 196 of 204 cases, axillary lymph node resection was performed and 62% of patients ($n$ = 121) were node positive and 38% ($n$ = 75) were node negative.

All carcinomas were invasive. Invasive ductal carcinomas accounted for 74% (145 of 204), lobular carcinomas for 17% (38 of 204) and invasive carcinomas of other types for 9% (20 of 204).

Tumours were graded according to Elston's grading system (Elston et al, 1991). Grade I tumours accounted for 22% (45 of 204), grade II for 54% (109 of 204) and grade III for 24% (49 of 204). Carcinomas were also graded according to Le Doussal's modified Bloom's grading system (Le Doussal et al, 1989) in five grades: 8% of grade I, 30% of grade II, 31% of grade III, 27% of grade IV and 4% of grade V. Tumours were also ranked according to the Nottingham prognostic index (Galea et al, 1992), which ranged from 2.1 to 8 (mean 4.1; s.d. 1.22). Tumours were aneuploid in 58% of the cases (97/168), with a variable degree of hyperploidy (mean 5.4%, range 0–60%) (cell imprints, Feulgen staining, image analysis) (Charpin et al, 1990, 1992).

### Immunohistological staining procedures

*Antibody sources*

Anti-human $VLA_2$ (150-kDa integrin α2 chain, anti-CD29b) mouse monoclonal (immunoglobulin G1) antibody (clone Gi9) was purchased from Immunotech (Marseille, France) and used diluted at 1:500 (Arihiro et al, 1993; Bonkhoff et al, 1993). The other monoclonal antibodies used were all commercially available

**Figure 1** $VLA_2$ expression in frozen section (anti-$VLA_2$, Immunotech) obtained using automated (Ventana) and quantitative (SAMBA) immunoperoxidase. Positive $VLA_2$ immunostaining is observed in epithelial cells of grade 2 breast ductal carcinomas

**Figure 2** $VLA_2$ expression in frozen section (anti-$VLA_2$ Immunotech) obtained using automated (Ventana) and quantitative (SAMBA) immunoperoxidase. Positive $VLA_2$ immunostaining is observed in epithelial cells of grade 3 breast ductal carcinomas

**Figure 3** $VLA_2$ expression in frozen section (anti-$VLA_2$ Immunotech) obtained using automated (Ventana) and quantitative (SAMBA) immunoperoxidase. Positive $VLA_2$ immunostaining is observed in tumour cells of lobular breast carcinomas and were used as described previously (Charpin et al, 1988*a*, 1994, 1995*b*, 1997*a–d*): MIBI, anti-CD31, anti-VCAM (clone 1Gl), ELAM (clone 1.2 B6), $VLA_2$ (clone Gi9) (Immunotech), anti-p53 (Oncogene Science, Paris, France), anti-c-erbB-2 (Biogenex Menarini, Chevilly Larue, France), anti-cathepsin D (CisBio International, Gif sur Yvette, France), anti-P-glycoprotein (P-gp) (clone JSBl-N, Tebu, Le Perray en Yvelines, France), anti-ER and -PR (Abbott kits, Rungis, France), anti-pS2 (Cisbio, France), anti-CD44 V6 (clone 2F10, RD System Europe, Abingdon, Oxon,

© Cancer Research Campaign 1998

**Figure 4** Distribution of positive $VLA_2$ surfaces evaluated (%) on tissue sections by computer-assisted (SAMBA) analysis of digitized microscopic coloured images

UK), anti-E cadherin (clone HECD1, RD System), anti-bcl-2 (clone 124) (Dako, 78196 Trappes, France).

### *Automated immunohistochemistry*

Automated immunohistochemistry was used for all the monoclonal antibodies, except for anti-E cadherin (LSAB kits, Dako) (Charpin et al, 1997*c*) and anti-ER and anti-PR (Abbott Kits) (Charpin et al, 1988*b*) and was performed on consecutive sections (4 µm thick) with avidin–biotin–peroxidase complex on Ventana 320 device (Grogan et al, 1993, 1995) (Ventana) Ventana kits (Ventana Systems, Strasbourg, France) including aminoethylcarbazol reagent. Sections were counterstained with haematoxylin, dehydrated and mounted in glycergel.

### *Image processing and statistical analysis*

Images of immunoprecipitates were obtained using an Axiophot microscope (Zeiss, Le Pecq 78230, France) and a 3CCD camera (Sony, Paris, France) and were then processed by an image analysis system (SAMBA 2005, Alcatel-TITN, Grenoble, France) (Charpin et al, 1997*a–d*). The two parameters of densitometric analysis, percentage of immunostained surface (compared with counterstained surface) and mean optical density (MOD), which reflects the staining intensity (on SAMBA arbitrary units scale of 0–255) were obtained as previously reported (Charpin et al, 1988*a* and *b*, 1994, 1995*a* and *b*, 1996*a* and *b*, 1997*a–d*). Statistical analysis was carried out using NCSS 6.01 statistical software (Kaysville, Utah, and Deltasoft, Meylan 38200, France). Various statistical tests were used depending on the type (nominal or ordinal) and distribution (normal or not) of the variables. Consequently, parametric or non-parametric tests were applied, including the chi-square test, Student's *t*-test, Kruskal–Wallis test, Mann–Whitney *U*-test and the calculation of correlation coefficients (Spearman's, Kendall's and Pearson's tests).

### RESULTS

### Patterns of $VLA_2$ distribution in cells and tissues

Patterns of immunoreaction were heterogeneous, as shown in Figures 1–3. Anti-$VLA_2$ reacted with epithelial cells but not in tumour stroma. The positive immunoreactions with anti-$VLA_2$ in ductal carcinomas were not significantly different from lobular carcinomas or from carcinomas of other types. In invasive ductal carcinomas (grade I–III), staining was variable, but variations in positive immunoreactions observed in individual tumour were independent of tumour dedifferentiation.

**Table 1** $VLA_2$ expression and clinicopathologic data

| | $VLA_2 = 0$ | $VLA_2 > 0$ | |
|---|---|---|---|
| Size (mm) | | | |
| ≤ 10 | 46 | 18 | $\chi^2 = 2.72$ |
| 11–20 | 68 | 17 | $P = 0.44$ (NS) |
| 21–30 | 2213 | 9 | |
| > 30 | | 2 | |
| < 15 | 85 | 29 | $\chi^2 = 0.52$ |
| ≥15 | 64 | 17 | $P = 0.47$ |
| Grade[a] | | | |
| SBR (Elston's) | | | |
| 1 | 26 | 16 | $\chi^2 = 4.78$ |
| 2 | 79 | 21 | $P = 0.09$ (NS) |
| 3 | 34 | 10 | |
| SBRM (Le Doussal's) | | | |
| 1 | 11 | 3 | |
| 2 | 41 | 16 | |
| 3 | 43 | 12 | $\chi^2 = 2.43$ |
| 4 | 38 | 7 | $P = 0.66$ (NS) |
| 5 | 5 | 2 | |
| Group I | 95 | 31 | $\chi^2 = 1.13$ |
| Group II | 43 | 9 | $P = 0.29$ (NS) |
| NPI (Galea) | | | |
| 1 (< 3.4) | 59 | 21 | $\chi^2 = 0.61$ |
| 2 (3.4–5.4) | 15 | 15 | $P = 0.74$ (NS) |
| 3 (> 5.4) | 18 | 4 | |
| Histological types | | | |
| Ductal | 110 | 38 | $\chi^2 = 0.71$ |
| Lobular | 37 | 9 | $P = 0.40$ (NS) |
| Node status | | | |
| Node positive | 93 | 28 | $\chi^2 = 0.45$ |
| Node negative | 55 | 20 | $P = 0.50$ (NS) |
| Feulgen staining | | | |
| Diploid | 49 | 16 | $\chi^2 = 0.02$ |
| Aneuploid | 69 | 23 | $P = 0.89$ |
| No hyperploidy | 21 | 6 | $\chi^2 = 0.12$ |
| Hyperploidy | 17 | 33 | $P = 0.73$ (NS) |

[a]Statistics calculated using the Mann–whitney test. SBR, Scarff–Bloom–Richardson score; SBRM, SBR modified.

### $VLA_2$ quantitative immunodetection

Only 23.5% (48 of 204) of the tumours were $VLA_2$ positive. The distribution of anti-$VLA_2$-positive staining as evaluated by densitometry on tissue sections is shown in Figure 4. Positive tumour surfaces stained by anti-$VLA_2$ ranged from 3% to 40% (mean 8.75%; s.d. 7.2).

### $VLA_2$ expression and clinicopathological data

$VLA_2$-immunostained surface evaluated by image analysis was independent of the patients' age, tumour size, histological type,

*© Cancer Research Campaign 1998*

**Table 2** $VLA_2$ immunocytochemical expression and other immunocytochemical markers in breast carcinomas

| Quantitative immunocytochemistry (positive surface) | VLA2 (all tumours) *n* = 203 | VLA2 = 0 (negative tumours) *n* = 155/203 | VLA2 > 0 (positive tumours) *n* = 48/203 | Statistical tests | |
|---|---|---|---|---|---|
| | | | | *t* | *P* |
| Ki 67/MIB1 (%) | | | | | |
| All tumours | 14.26 (11.30) | 15.11 (11.84) | 11.54 (8.96) | 2.22 | 0.034 |
| Positive tumours | 14.26 (11.30) | 15.11 (11.84) | 11.54 (8.96) | 2.22 | 0.034 |
| c-erbB-2 (%) | | | | | |
| All tumours | 18.85 (15.18) | 18.56 (15.92) | 19.82 (12.48) | 0.51 | 0.615 |
| Positive tumours | 19.43 (15.04) | 19.16 (15.82) | 20.35 (12.20) | 0.48 | 0.632 |
| p53 (%) | | | | | |
| All tumours | 4.22 (11.86) | 4.79 (13.11) | 2.40 (6.07) | 1.75 | 0.091 |
| Positive tumours | 18.23 (18.89) | 21.20 (20.51) | 9.58 (9.06) | 2.68 | 0.012 |
| bcl-2 (%) | | | | | |
| All tumours | 17.31 (17.40) | 17.10 (17.43) | 17.98 (17.46) | 0.30 | 0.763 |
| Positive tumours | 22.21 (16.72) | 21.01 (17.06) | 26.97 (14.55) | 2.00 | 0.055 |
| E cadherin (%) | | | | | |
| All tumours | 30.23 (18.28) | 29.71 (18.58) | 31.97 (17.38) | 0.75 | 0.460 |
| Positive tumours | 33.33 (16.27) | 33.11 (16.48) | 34.02 (15.76) | 0.33 | 0.740 |
| CD44 v6 (%) | | | | | |
| All tumours | 14.04 (14.62) | 13.66 (14.93) | 15.27 (13.63) | 0.70 | 0.490 |
| Positive tumours | 17.05 (14.43) | 16.78 (14.88) | 17.88 (13.06) | 0.45 | 0.654 |
| CD31 (%) | | | | | |
| All tumours | 10.95 (6.55) | 11.18 (6.55) | 10.21 (6.58) | 0.88 | 0.386 |
| Positive tumours | 11.01 (6.52) | 11.25 (6.51) | 10.21 (6.58) | 0.95 | 0.351 |
| Cathepsin D (%) | | | | | |
| All tumours | 19.3 (11.7) | 20.9 (12.4) | 17.6 (11.2) | 1.44 | 0.161 |
| Positive tumours | 29.3 (14.3) | 30.9 (17.5) | 24.3 (15.2) | 1.44 | 0.161 |
| P-gp (%) | | | | | |
| All tumours | 2.52 (4.16) | 2.19 (3.66) | 3.56 (5.37) | 1.65 | 0.109 |
| Positive tumours | 6.21 (4.44) | 5.93 (3.76) | 6.84 (5.75) | 0.73 | 0.473 |
| ER (%) | | | | | |
| All tumours | 11.71 (14367) | 12.31 (15.11) | 9.77 (11.52) | 1.23 | 0.227 |
| Positive tumours | 16.98 (14.48) | 18.00 (15.22) | 13.79 (11.48) | 1.71 | 0.098 |
| PR (%) | | | | | |
| All tumours | 11.40 (15.12) | 11.15 (15.14) | 12.23 (15.17) | 0.43 | 0.669 |
| Positive tumours | 18.52 (15.47) | 18.19 (15.69) | 19.57 (14.98) | 0.43 | 0.667 |
| pS2 (%) | | | | | |
| All tumours | 3.76 (6.32) | 3.66 (6.44) | 4.08 (5.97) | 0.42 | 0.677 |
| Positive tumours | 8.69 (7.04) | 9.33 (7.28) | 7.26 (6.36) | 1.34 | 0.189 |
| VCAM (%) | | | | | |
| All tumours | 2.59 (4.57) | 1.62 (2.78) | 5.70 (7.16) | 3.85 | 0.001 |
| Positive tumours | 6.33 (5.24) | 4.66 (2.85) | 9.43 (7.04) | 3.50 | 0.001 |
| $VLA_3$ (%) | | | | | |
| All tumours | 8.18 (11.16) | 6.40 (8.97) | 13.38 (14.89) | 2.69 | 0.012 |
| Positive tumours | 13.79 (11.51) | 11.33 (9.31) | 19.80 (14.14) | 2.76 | 0.010 |
| ELAM (%) | | | | | |
| All tumours | 6.54 (8.73) | 5.19 (7.12) | 10.92 (11.65) | 3.23 | 0.003 |
| Positive tumours | 11.758 (8.69) | 10.31 (6.92) | 14.97 (11.18) | 2.28 | 0.030 |

Numbers in parentheses are standard deviation values.

tumour differentiation and ploidy, histoprognostic grades, lymph node status and Nottingham prognostic index. No significant difference between these parameters and anti-$VLA_2$-positive or -negative reaction was observed. Moreover, there was no significant relationship between $VLA_2$ expression evaluated by quantitative immunocytochemistry at various cut-off points (5%, 10%, 15%) and any of the clinicopathological parameters studied (Table 1).

### Correlation of $VLA_2$ expression with quantitative immunodetection

### *Distribution*

The distribution of the antigens, investigated using the same method as that for $VLA_2$ on serial sections, was first studied by calculation of the mean positive (immunostained) surface (%) in $VLA_2$-positive (48 of 204) and -negative (156 of 204) subsets.

The results of quantitative evaluation of growth fraction (MIB1) and immunoreaction with anti-p53, anti-bcl-2, anti-c-erbB-2 protein, anti-cathepsin D, anti-CD31, anti-CD44v, anti-cadherin, anti-VCAM, anti-ELAM, anti-$VLA_3$, anti-P-gp, anti-ER and -PR antigenic sites, and anti-pS2 in $VLA_2$ -positive and -negative tumours are shown in Table 2.

### *Correlation (Spearman's and Kendall's tests)*

$VLA_2$ expression in breast carcinomas was independent of most of the other antigens investigated (Table 3). However, a significant correlation was observed between $VLA_2$ immunohistochemical expression and that of P-gp, VCAM, ELAM and $VLA_3$ (Tables 3 and 4) and was inversely correlated with cathepsin D.

© Cancer Research Campaign 1998 

Table 3 Correlation of quantitative immunocytochemical assays of $VLA_2$ to that of other antigens investigated on consecutive sections of frozen blocks, automated (Ventana) procedure and image analysis (SAMBA)

| | $VLA_2$ immunocytochemical expression | |
|---|---|---|
| Cathepsin D | $r = -0.298$ | $P < 0.001$ (Spearman) |
| ELAM | $r = 0.345$ | $P < 0.01$ (Spearman) |
| VCAM | $r = 0.499$ | $P < 0.001$ (Spearman) |
| $VLA_3$ | $r = 0.193$ | $P < 0.01$ (Spearman) |
| P-gp | $r = 0.425$ | $P = 0.036$ (Spearman) |
| Ki67/MIB1 | | NS |
| p53 | | NS |
| bcl-2 | | NS |
| c-erbB-2 | | NS |
| E cadherin | | NS |
| CD44v | | NS |
| CD31 | | NS |
| ER | | NS |
| PR | | NS |
| pS2 | | NS |

### DISCUSSION

Mortality in breast cancer is related to the development of blood-borne metastases. Tumour metastases require the release of cells from the primary site, migration through the extracellular matrix into the microvasculature and finally arrest in distant organ (Zutter and Santoro, 1990). Various molecules, especially adhesion molecules, are involved at the different steps of the metastatic process and are expressed on tumour cells or released in the extracellular compartment (Zutter and Santoro, 1990; Albelda, 1993; Pignatelli and Vessey, 1994). Thus their immunodetection in tissue sections may be a relevant tool useful in clinical practice to identify patients with significant risk of developing metastatic disease, promoted by the variations in the expression of the adhesive molecules. However, the practical relevance of immunohistochemistry can be determined provided that the technical procedures are as far as possible standardized and provided that methodological bias are optimally reduced.

Methodological bias may result from several factors. Firstly, tissue fixation and paraffin embedding may be the source of antigen damage. Although recent advances in immunohistochemistry have shown the suitability of archival paraffin tissue blocks for immunodetection after antigen retrieval (microwave), the freezing of tissue blocks, when possible, basically does not modify the structure of antigens in tissues. Secondly, inconsistencies may result from variations in the immunohistochemical methods used. In this regard, an automated device rather than in-house manual procedures provides a better guarantee of quality control, particularly with regard to reproducibility. Finally, analytical bias may result from the method used for the evaluation of the results. Semiquantitative analysis of the immunoprecipitates in tissue sections is convenient, rapid and cost effective, but its reliability hinges on observers' experience and reproducibility. Although semiquantitative evaluation is obviously sufficient to differentiate negative vs positive reaction, it is not accurate enough to evaluate intermediate patterns of staining. In contrast, quantitative analysis by computerized processing of digitized microscopic images provides more objective data, and variations in staining are more accurately evaluated. In addition, the numeric values of parameters are more suitable for statistical analysis. In the present study, we evaluated $VLA_2$ in optimal technical conditions using frozen tissue sections, automated immunohistochemistry and computerized analysis of digitized microscopic images, as previously used for other molecules (Charpin et al, 1988*a* and *b*, 1994, 1995*a* and *b*, 1996*b*, 1997*a–d*).

Adhesion molecules are involved in the cascade of events of the metastatic disease. Some are involved in cell–cell adhesion or in cell adhesion to epithelial basement membrane. Some are involved in adhesion to extracellular matrix and others in adhesion to the vessel wall either to activate endothelial cells or to increase binding to vessel basement membrane. $VLA_2$, which is an $\alpha 2\ \beta 1$ integrin acting as a receptor for collagen and laminin, is involved in cell binding to epithelial basement membrane and also to the vessel basement membrane. These adhesive properties of tumour cells expressing $VLA_2$ promote the development of metastases as shown by experimental data (Chan et al, 1991) and facilitate adhesion to the vessel wall. However, the decreased expression of $VLA_2$ in human carcinomas (Zutter et al, 1990); Pignatelli et al, 1991*a* and *b*; Stamp and Pignatelli, 1991; Arihiro et al, 1993; Bonkhoff et al, 1993; Patriarca et al, 1993) is associated with higher metastatic rate or tumour dedifferentiation. In this respect, the $VLA_2$ expression of tumour cells in early breast carcinomas could constitute a prognostic indicator.

Using optimal technical conditions (not comparable to previous studies for $VLA_2$ immunodetection) in our series of breast carcinomas, we did not observe any significant relationship between $VLA_2$ expression and current histoprognostic indicators, such as tumour size, grade, type, degree of tumour aneuploidy and hyperploidy, or the axillary node status and the Nottingham prognostic index. These results suggest that in order to determine its exact prognostic relevance, the significance of $VLA_2$ expression in

Table 4 $VLA_2$ immunocytochemical expression and Cathepsin D in breast carcinomas

| | $VLA_2 \leq 5\%$ | $5\% < VLA_2 \leq 10\%$ | $10\% < VLA_2 \leq 20\%$ | $VLA_2 > 20\%$ | Total | Chi-square test |
|---|---|---|---|---|---|---|
| Cathepsin D ≤ 15% | 83 | 6 | 6 | 0 | 95 | |
| Percentage of line | 87.3 | 6.3 | 6.3 | 0 | | |
| Percentage of column | 51.6 | 24 | 60 | 0 | | |
| 15% < Cathepsin D < 20% | 25 | 9 | 1 | 0 | 35 | $\chi^2 = 13.43$ |
| Percentage of line | 71.4 | 25.7 | 2.9 | 0 | | $P = 0.037$ |
| Percentage of column | 15.5 | 36 | 10 | 0 | | |
| Cathepsin D < 20% | 53 | 10 | 3 | 2 | 68 | |
| Percentage of line | 77.9 | 14.7 | 4.4 | 2.9 | | |
| Percentage of column | 32.9 | 40 | 30 | 100 | | |

 © Cancer Research Campaign 1998

breast carcinomas requires further investigation, using the same method, taking into account correlation with the patients' outcome.

The adhesive properties of tumour cells probably depend on the expression of adhesion molecules, which may vary independently or concomitantly. $VLA_2$ expression by tumour in breast carcinomas seems to be independent of cell–cell adhesion or adhesion to extracellular matrix, as $VLA_2$ immunostaining was independent of E cadherin or of CD44 v6 expression detected using the same method (Charpin et al, 1997*b* and *c*). However, $VLA_2$ significantly correlated with $VLA_3$ ($P < 0.01$), a receptor for fibronectin that also belongs to the integrin group and that may have similar regulation mechanisms.

We found that $VLA_2$ expression was inversely correlated with cathepsin D (Spyratos et al, 1989) expression in tumour sections, suggesting that the decreased capacity of tumour cells to bind to basement membrane is associated with an increased capacity of tumour cell migration in the extracellular matrix, facilitated by proteases that they produce. Thus, $VLA_2$ and D cathepsin seem to concomitantly promote tumour progression.

Angiogenesis in breast carcinomas favours tumour growth and facilitates entry into the circulation (Folkman, 1971; Liotta et al, 1974), and vessel counts and immunohistochemical labelling of vessels and endothelial cells have been shown to be endowed with some clinical relevance (Weidner, 1991; Charpin et al, 1997*a*). Our results show that $VLA_2$ immunocytochemical expression is independent of that of CD31, which reflects tumour stromal angiogenesis (Charpin et al, 1995*b*). However, $VLA_2$ was found to correlate ($P < 0.01$) with the expression of VCAM (IGSF) and ELAM (E-selectin) in tumours reflecting the activation of endothelial cells. This suggests that the increased expression of ELAM or VCAM molecules, which are receptors for ligands expressed on the surface of tumour cells, such as Sialyl Lewis$^x$ determinants (Wälz et al, 1990; Tiemeyer et al, 1991) or $VLA_4$ (Albelda et al, 1990; Elices et al, 1990; Gelhsen et al, 1992), correlates with the capacity of tumour cells, to bind vessel basement membranes through $VLA_2$ expression.

Some experimental studies have shown that collagen-induced morphogenesis and expression of $\alpha2$ integrin subunit is inhibited in c-*erb*B-2-transfected MTSV1-7 human mammary epithelial cells (D'Souza et al, 1993), suggesting that in human breast carcinomas the expression of c-*erb*B-2 product could correlate with integrin expression. However, in our study, $VLA_2$ / $\alpha2\ \beta1$ integrin did not significantly correlate with c-erbB-2, both molecules being detected according to the same immunohistochemical procedures on consecutive sections.

Recently, we also used this method to detect p53, bcl-2 and Ki67 antigens in breast carcinomas (Charpin et al, 1996*a*, 1997*b* and *d*). These antigens have been reported to correlate with tumour growth and tumour cell proliferative activity. Similar to C-erbB2 expression, the expression of these molecules did not correlate with $VLA_2$ expression, suggesting that cell proliferation and adhesive properties are regulated differently.

In conclusion, our results show that $VLA_2$ immunohistochemical expression in breast carcinomas is independent of most current histoprognostic indicators and of the tumour cells expression of some adhesion molecules, such as E cadherins. However, $VLA_2$ expression correlates with the expression in tumours of adhesion molecules of the same family, such as $VLA_3$ integrin or another family, such as E-selectin (ELAM) or IGSF (VCAM).

The prognostic significance of $VLA_2$ expression in tumours remains to be demonstrated by correlations with the patients' outcome.

### ACKNOWLEDGEMENTS

This study was supported by grants from la Fondation pour la Recherche Médicale and Aix Marseille II University (BQR 1997).

### REFERENCES

Albelda SM (1993) Role of integrins and other cell adhesion molecules in tumor progression and metastasis. *Lab Invest* **68**: 4–17

Arihiro K, Inai K, Kurihara K, Takeda S, Kaneko M, Kuroi K and Toge T (1993) Loss of VLA-2 collagen receptor in breast carcinoma, facilitating invasion and metastasis. *Jpn J Cancer* **84**: 726–733

Bonkhoff H, Stein U and Remberger K (1993) Differential expression of α6 and α2 very late antigen integrins in the normal, hyperplastic, and neoplastic prostate: simultaneous demonstration of cell surface receptors and their extracellular ligands. *Hum Pathol* **24**: 243–248

Brugal G, Garbay C, Giroud-Adhel D (1979) A double scanning microphotometer for image analysis: hardware, software and biomedical application. *J Histochem Cytochem* **27**: 144

Chan BMC, Matsuura N, Takada Y, Zetter BR and Hemler ME (1991) In vitro and in vivo consequences of VLA-2 expression on rhabdomyosarcoma cells. *Science* **251**: 1600–1602

Charpin C, Andrac L, Vacheret H, Habib MC, Devictor B, Lavaut MN, Toga M (1988*a*) Multiparametric evaluation (SAMBA) of growth fraction (monoclonal Ki67) in breast carcinoma tissue sections. *Cancer Res* **48**: 4368–4374

Charpin C, Martin PM, Devictor B, Lavaut MN, Habib MC and Toga M (1988*b*) Multiparametric study (SAMBA 200) of estrogen receptor immunocytochemical assay in 400 human breast carcinomas analysis of estrogen receptor distribution heterogeneity in tissues and correlations with dextran coated charcoal assays and morphological data. *Cancer Res* **48**: 1578–1586

Charpin C, Andrac L, Lavaut MN, Andonian C, Fraterno M, Devictor B, Perez-Castillo A, Bonnier P and Piana L (1990) Image cytometry of aneuploidy, growth fraction (MoAb Ki-67) and hormone receptors (ER, PR) immunocytochemical assays in breast carcinomas. *Anal Cell Pathol* **2**: 357–371

Charpin C, Bonnier P, Piana L, Kouzhami H, Devictor B, Lavaut MN, Andrac L and Allasia C (1992) Correlation of nuclear organizer regions and nuclear morphometry assessed by automatic image analysis in breast cancer with aneuploidy, Ki67 immunostaining, histopathologic grade and lymph node involvement. *Pathol Res Pract* **188**: 1009–1017

Charpin C, Vielh P, Duffaud F, Devictor B, Andrac L, Lavaut MN, Allasia C, Horschowski N and Piana L (1994) Quantitative immunocytochemical assays of P-glycoprotein in breast carcinomas: correlation to messenger RNA expression and to immunohistochemical prognostic indicators. *J Natl Cancer Inst* **86**: 1539–1545

Charpin C, Devictor B, Andrac L, Amabile J, Bergeret D, Lavaut MN, Allasia C and Piana L (1995*a*) p53 quantitative immunocytochemical analysis in breast carcinomas. *Hum Pathol* **26**: 159–166

Charpin C, Devictor B, Bergeret D, Andrac L, Boulat J, Horschowski N, Lavaut MN and Piana L (1995*b*) CD31 quantitative immunocytochemical assays in breast carcinomas: correlation with current prognostic factors. *Am J Clin Pathol* **103**: 443–448

Charpin C, Garcia S, Bouvier C, Devictor B, Andrac L, Lavaut MN, Allasia C and Bonnier P (1996*a*) Prognostic value of Ki 67/MIB1 automated and quantitative immunolabelling in primary operable breast carcinomas. *Int J Oncol* **9**: 337–344

Charpin C, Garcia S, Bouvier C, Andrac L, Devictor B, Lavaut MN, Allasia C, Bonnier P, Piana L (1996*b*) Quantitative immunocytochemical assays on frozen sections of p53. Correlation to the follow-up of patients with breast carcinomas. *Am J Clin Pathol* **106**: 640–646

Charpin C, Garcia S, Bouvier C, Martini F, Andrac L, Bonnier P, Lavaut MN, Allasia C (1997*a*) CD31/PECAM automated and quantitative immunocytochemical assays in breast carcinomas. Correlation with patient follow-up. *Am J Clin Pathol* **107**: 534–541

Charpin C, Garcia S, Bouvier C, Devictor B, Andrac L, Choux R, Lavaut MN and Allasia C (1997*b*) Automated and quantitative immunocytochemical assays of CD44v6 in breast carcinomas. *Hum Pathol* **28**: 289–296

Charpin C, Garcia S, Bouvier C, Devictor B, Andrac L, Choux R and Lavaut MN (1997*c*) E-Cadherin quantitative immunocytochemical assays in breast carcinomas. *J Pathol* **181**: 294–300

© Cancer Research Campaign 1998

Charpin C, Garcia S, Bouvier C, Devictor B, Andrac L, Lavaut MN and Allasia C (1997*d*) Automated and quantitative immunocytochemical assays of Bcl-2 protein in breast carcinomas. *Br J Cancer* **76**: 340–346

De Strooper B, Van der Schuerer D, Jaspers M, Saison M, Spaepen M, Val Leuven F, Van Den Berghe H and Cassiman JJ (1989) Distribution of the β1 subgroup of the integrins in human cells and tissues. *J Histochem Cytochem* **37**: 299–307

D'Souza B, Berdichevsky F, Kyprianou N and Taylor-Papadimitriou J (1993) Collagen-induced morphogenesis and expression in the α2-integrin subunit is inhibited in c-erb B2-transfected human mammary epithelial cells. *Oncogene* **8**: 1797–1806

Elices MJ, Osborn L, Takada Y, Crouse C, Luhowski JS, Hemler ME and Lobb RR (1990) VCAM-1 on activated endothelium interacts with the leucocyte integrin VLA-4 at a site distinct from the VLA-4/fibronectin binding site. *Cell* **60**: 577–584

Elston CW and Ellis IO (1991) Pathological prognostic factors in breast cancer. I. The value of histological grade in breast cancer: experience from a large study with long term follow-up. *Histopathology* **19**: 403–410

Folkman J (1971) Tumor angiogenesis: therapeutic implications. *N Engl J Med* **285**: 1182–1186

Galea MH, Blamey RW and Elston CE (1992) The Nottingham prognostic index in primary breast cancer. *Breast Cancer Res Treat* **22**: 207–219

Gehlsen KR, Davis GE and Sriramatao P (1992) Integrin expression in human melanoma cells with differing invasive and metastatic properties. *Clin Exp Metast* **10**: 111–120

Grogan TM, Casey TT, Miller PC, Rangel C, Nunnery D and Nagle R (1993) Automation of immunohistochemistry. *Adv Pathol Lab Med* **6**: 253–283

Grogan TM, Rangel C, Rimsza L, Bellamy W, Martel R, McDaniel D, McGraw B, Richards W, Richards L, Rodgers P, Rybski J, Showalter W and Zeheb R (1995) Kinetic-mode, automated double-labeled immunohistochemistry and in situ hybridization in diagnostic pathology. *Adv Pathol Lab Med* **8**: 79–99

Hynes RO (1987) Integrins, a family of cell surface receptors. *Cell* **48**: 549–555

Le Doussal V, Tubiana-Hulin M, Friedman S, Hacene K, Spyratos F and Brunet M (1989) Prognostic value of histologic grade nuclear components of Scarff–Bloom–Richardson (SBR): an improved score modification based on a multivariate analysis of 1262 invasive ductal breast carcinomas. *Cancer* **64**: 1914–1921

Liotta LA, Kleinerman J and Saidel GM (1974) Quantitative relationships of intravascular tumor cells, tumors vessels, and pulmonary metastases following tumor implantation. *Cancer Res* **34**: 997–1004

Patriarca C, Roncalli M, Gambacorta M, Cominotti M, Coggi G and Viale G (1993) Patterns of integrin common chain β1 and collagen IV immunoreactivity in hepatocellular carcinoma correlations with tumour growth rate, grade and size. *J Pathol* **5**: 5–11

Pignatelli M and Vessey CJ (1994) Adhesion molecules: novel molecular tools in tumor pathology. *Hum Pathol* **25**: 849–856

Pignatelli M, Smith MEF and Bodmer WF (1991*a*) Low expression of collagen receptors in moderate and poorly differentiated colorectal adenocarcinomas. *Br J Cancer* **61**: 636–638

Pignatelli M, Hanby AM and Stamp GWH (1991*b*) Low expression of β1, α2 and α3 subunits of VLA integrins in malignant mammary tumors. *J Pathol* **165**: 25–32

Ruoslahti E (1991) Integrins. *J Clin Invest* **87**: 1–5

Schadendorf D, Gawlik C, Haney U, Ostmeier H, Suter L, Czarnetzki B (1993) Tumour progression and metastatic behaviour in vivo correlates with integrin expression on melanocytic tumours. *J Pathol* **170**: 429–434

Spyratos F, Brouillet JP, Defrenne A, Hacene K, Rouesse J, Maudelonde T, Brunet M, Andrieu C, Desplaces A and Rochefort H (1989) Cathepsin D: an independent prognostic factor for metastasis of breast cancer. *Lancet* **8672**: 1115–1118

Stamp GWH and Pignatelli M (1991) Distribution of β1, α1, α2 and α3 integrin chains in basal cell carcinomas. *J Pathol* **163**: 307–313

Tiemeyer M, Swiedler SJ, Ishihara M, Moreland M, Schweingruber H, Hirtzer P and Brandley BK (1991) Carbohydrate ligands for endothelial-leukocyte adhesion molecule 1. *Proc Natl Acad Sci* USA **88**: 1138–1142

Walz G, Aruffo A, Kolanus W, Bevilacqua M and Seed B (1990) Recognition by ELAM-1 of the Sialyl-Le$^x$ determinant on myeloid and tumor cells. *Science* **250**: 1132–1135

Weidner N, Semple JP, Welch WR and Folkman J (1991) Tumor angiogenesis and metastasis: correlation in invasive breast carcinoma. *N Engl J Med* **324**: 1–8

Zetter BR (1993) Adhesion molecules in tumor metastasis. *Cancer Biology* **4**: 219–229

Zutter MM and Santoro SA (1990) Widespread histologic distribution of the α2β1 integrin cell surface collagen receptor. *Am J Pathol* **137**: 113–120

Zutter MM, Mazoujian G and Santoro SA (1990) Decreased expression of integrin adhesive protein receptors in adenocarcinoma of the breast. *Am J Pathol* **137**: 863–870

© Cancer Research Campaign 1998